# 76. Uncomplicated Urinary Tract Infections in the Multi-resistance Era: Is It Time of Microbiologic Diagnosis? An Observational Study in Buenos Aires City

**DOI:** 10.1093/ofid/ofab466.278

**Published:** 2021-12-04

**Authors:** Alvaro Otreras, Sofia Sabato, Daniela D′Alessandro, Sylvia Errea, Edgardo Bottaro, Cecilia Ormazabal, Claudia Alfonso, Pablo Scapellato

**Affiliations:** Hospital Donación Francisco Santojanni, Ciudad Autonoma de Buenos Aires, Ciudad Autonoma de Buenos Aires, Argentina

## Abstract

**Background:**

Uncomplicated urinary tract infection (uUTI) is one of the main causes of antibiotics prescription in outpatient setting. Current recommendations, based on studies from pre-antimicrobial resistance era, suggest that diagnosis of uUTI can be made based on clinical symptoms and that urine analysis leads only to a minimal increase in diagnostic accuracy. We analyzed urine cultures (UC) from patients with clinical diagnosis.

**Methods:**

Prospective and observational study carried out in an Emergency Department during August 2016 to August 2017. Women older than 15 years with 2 or more classic symptoms of uUTI and the absence of vaginal discharge and irritation were included. Those with complicated and recurrent urinary tract infection (UTI) were excluded. Urine cytology and UC were performed in all episodes. A bivariate and multivariate analysis was performed considering the probability of having a positive urine culture according to the different symptomatology variables.

**Results:**

We enrolled 208 patients, with a median age of 25 (14-68 years). Previous UTI 6 (2.9%), previous antibiotic (last 3 months) 20 (9.6%). Inflammatory cytology 173 (83.2%), positive UC 109 (52.4%), cystitis 155 (74.5%). Symptoms: dysuria 154 (74%), frequency 111 (53.4%), tenesmus 97 (46.6%), fever 78 (37.5%), hematuria 43 (20, 7%), hypogastric pain 128 (61.5%), back pain 84 (40.4%). Combinations of 3 or more classic symptoms occurred in 52 (25%) episodes. The most frequent association was dysuria, frequency and tenesmus. No statistically significant association was found either in the bivariate or multivariate analysis in relation to presenting positive UC (Tables 1 and 2).

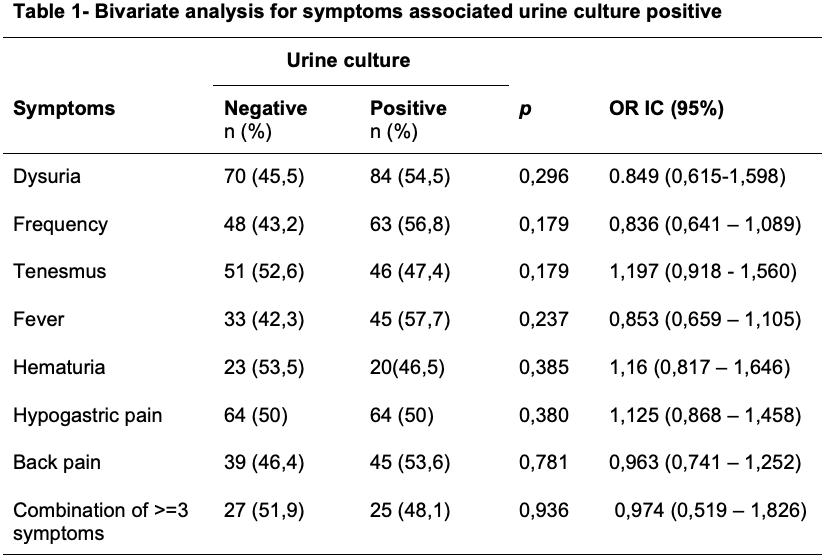

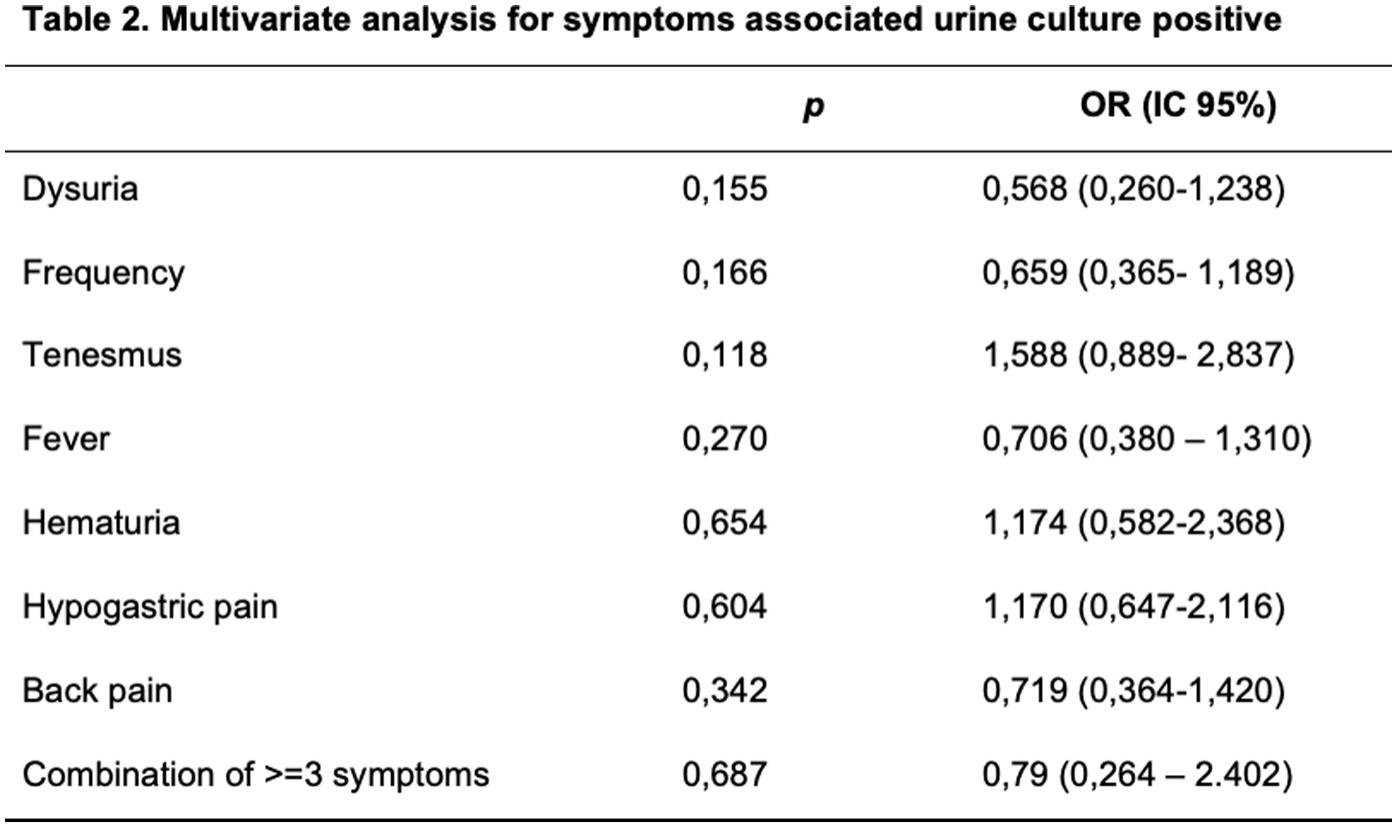

**Conclusion:**

The results show that almost 50% of the patients with a clinical diagnosis of UTI had a negative urine culture. We consider it necessary to rethink the prescription of antibiotics without microbiological confirmation in the first episode of uUTI as a strategy to reduce inappropriate use of antibiotics.

**Disclosures:**

**All Authors**: No reported disclosures

